# High uptake of ^68^Ga-PSMA and ^18^F-DCFPyL in the peritumoral area of rat gliomas due to activated astrocytes

**DOI:** 10.1186/s13550-020-00642-0

**Published:** 2020-05-25

**Authors:** Dennis Oliveira, Carina Stegmayr, Alexander Heinzel, Johannes Ermert, Bernd Neumaier, N. Jon Shah, Felix M. Mottaghy, Karl-Josef Langen, Antje Willuweit

**Affiliations:** 1 Institute of Neuroscience and Medicine (INM), Forschungszentrum Jülich, D-52425 Jülich, Germany; 2 Department of Nuclear Medicine, RWTH Aachen University, Aachen, Germany; 3 Jülich-Aachen Research Alliance (JARA)–Section JARA-Brain, Aachen, Germany; 4 Department of Neurology, RWTH Aachen University, Aachen, Germany; 5 Department of Radiology and Nuclear Medicine, Maastricht University Medical Center (MUMC+), Maastricht, The Netherlands

## Abstract

**Background:**

Recent studies reported on high uptake of the PSMA ligands [^68^Ga]HBED-CC (^68^Ga-PSMA) and ^18^F-DCFPyL in cerebral gliomas. This study explores the regional uptake and cellular targets of ^68^Ga-PSMA and ^18^F-DCFPyL in three different rat glioma models.

**Methods:**

F98, 9 L, or U87 rat gliomas were implanted into the brains of 38 rats. After 13 days of tumor growth, ^68^Ga-PSMA (*n* = 21) or ^18^F-DCFPyL (*n* = 17) was injected intravenously, and animals were sacrificed 40 min later. Five animals for each tracer and tumor model were additionally investigated by micro-PET at 20–40 min post injection. Cryosections of the tumor bearing brains were analyzed by ex vivo autoradiography and immunofluorescence staining for blood vessels, microglia, astrocytes, and presence of PSMA. Blood-brain barrier (BBB) permeability was tested by coinjection of Evans blue dye (EBD). ^68^Ga-PSMA uptake after restoration of BBB integrity by treatment with dexamethasone (Dex) was evaluated in four animals with U87 gliomas. Competition experiments using the PSMA-receptor inhibitor 2-(phosphonomethyl)pentane-1,5-dioic acid (PMPA) were performed for both tracers in two animals each.

**Results:**

Autoradiography demonstrated a strong ^68^Ga-PSMA and ^18^F-DCFPyL binding in the peritumoral area and moderate binding in the center of the tumors. PMPA administration led to complete inhibition of ^68^Ga-PSMA and ^18^F-DCFPyL binding in the peritumoral region. Restoration of BBB by Dex treatment reduced EBD extravasation but ^68^Ga-PSMA binding remained unchanged. Expression of activated microglia (CD11b) was low in the intra- and peritumoral area but GFAP staining revealed strong activation of astrocytes in congruency to the tracer binding in the peritumoral area. All tumors were visualized in micro PET, showing a lower tumor/brain contrast with ^68^Ga-PSMA than with ^18^F-DCFPyL.

**Conclusions:**

High uptake of ^68^Ga-PSMA and ^18^F-DCFPyL in the peritumoral area of all glioma models is presumably caused by activated astrocytes. This may represent a limitation for the clinical application of PSMA ligands in gliomas.

## Introduction

PET ligands for the prostate specific membrane antigen (PSMA) are very successful in the diagnostic assessment of prostate cancer [1, 2]. The most widely studied agent is the ^68^Ga-labeled PSMA inhibitor Glu-NH-CO-NH-Lys(Ahx)-HBED-CC (^68^Ga-PSMA) but in recent years, ^18^F-labeled ligands such as 2-(3-(1-carboxy-5-[(6-[^18^F]fluoro-pyridine-3-carbonyl)-amino]-pentyl)-ureido)-pentanedioic acid (^18^F-DCFPyL) have been developed which show advantages with respect to production amount, availability, clinical utility, and image resolution [3, 4]. PSMA is also expressed by a variety of non-prostate cancers, often on the endothelium of tumor-associated neovasculature [5], and initial studies have explored the application of PSMA ligands in breast, lung, bladder, pancreatic and colorectal cancer, renal cell carcinoma, and glioblastoma [6]. In a triple negative breast cancer xenograft, the localization of PSMA was detected in the xenograft-associated endothelial cells as well as on the tumor cells [7]. Also, benign tissue like the cervical ganglia possesses a very strong PSMA expression [8].

Most of the reported findings with PSMA-targeted radiotracers in non-prostate malignancies, however, are limited to small series of patients, and further investigations are needed to explore the potential of PSMA ligands outside prostate cancer.

Glioblastoma is the most frequent malignant adult brain tumor and has a dismal prognosis. Various approaches have focused on the application of radionuclides for targeted therapy of these tumors [9]. Previous histopathological studies of human glioblastoma have identified PSMA on neovasculature of gliomas as a potential target for treatment [10, 11]. Subsequent pilot studies have reported on high uptake of ^68^Ga-PSMA and ^18^F-DCFPyL in brain tumors suggesting a potential role of PSMA ligands in theranostics [12–15]. It remains unclear, however, whether the binding of the PSMA ligands occurs mainly on tumor cells or endothelial cells and to what extent the accumulation is influenced by other factors such as disturbance of the blood-brain barrier (BBB). Furthermore, it is unknown to what extent the ligands bind to activated microglia and astroglia which are the two major types of glial cells involved in the regulation of the immune response to pathological processes in the brain and which could affect the specificity of the tracers for tumor detection.

In this study, we explored the regional binding of the well-established PSMA ligands ^68^Ga-PSMA and ^18^F-DCFPyL in three different rat glioma models including the human U87 glioma cell line. Special attention was paid to the evaluation of the role of microglia and astroglia, the role of BBB permeability, which was modulated by treatment with dexamethasone (Dex), and the specificity of tracer binding which was assessed by competition experiments with the PSMA-receptor inhibitor PMPA. The experiments provided interesting new insight into the cellular targets that might be involved in the accumulation of PSMA ligands in cerebral gliomas.

## Material and methods

### Animals

Thirty-eight male rats (Charles River Laboratories, Sulzfeld, Germany) were included in this study. An overview of the experiments, the rat strains, glioma models, tracers, pharmacological interventions, and number of animals in each group is given in Table 1. All animals were handled in accordance with the Animal Research Committee of the Forschungszentrum Jülich GmbH, the German Animal Welfare Act and the European Community Council directives regarding the protection of animals used for experimental and scientific purposes (2010/63/EU) and with the approval by the local ethics committee (Landesamt für Natur, Umwelt und Verbraucherschutz, North-Rhine-Westphalia, Germany, Az 84-02.04.2016.248). All rats weighted between 230 and 330 g and were housed in groups of two under standard conditions. Food and water were provided ad libitum.

**Table 1:** Overview of the experiments: glioma models, tracers, pharmacological interventions, and number of animals in each group

Rat strain	Glioma model	Tracer/treatment	No. of animals
Fischer 344	9 L	^68^Ga-PSMA	5
		^18^F-DCFPyL	5
		^18^F-DCFPyL + PMPA	2
	F98	^68^Ga-PSMA	5
		^18^F-DCFPyL	5
RNU	U87	^68^Ga-PSMA	5
		^18^F-DCFPyL	5
		^68^Ga-PSMA + PMPA	2
		^68^Ga-PSMA + Dex	4

### Cell culture and tumor inoculation

U87 and 9 L tumor cells (U87: ATCC® HTB-14™, LGC Standards GmbH; 9 L: ECACC GS-9 L, Salisbury, UK) were cultured in Minimum Essential Medium Eagle (MEM) supplemented with 10% fetal calf serum, 1% glutamine, 1% penicillin/streptomycin, and 1% non-essential amino acids. F98 tumor cells were cultured similar to U87 and 9 L, but in Dulbecco’s Modified Eagle’s Medium (DMEM) instead of MEM and without non-essential amino acids. After reaching about 95% of confluency, cells were prepared for inoculation into rat brains. Therefore, cells were washed with PBS and detached by incubation with a trypsin/EDTA solution for 5 min. Afterwards, cells were resuspended in cell medium generating a cell concentration of 500.000 cells/5 μl for the U87 cells, 65.000/5 μl for the 9 L cells, and 30.000/5 μl for the F98 cells.

Cell suspensions were then stereotactically inoculated into the left anterior striatum under anesthesia as described previously [16]. The tumor was allowed to grow for 13 days.

### Autoradiography and micro-PET

^18^F-DCFPyL and ^68^Ga-PSMA were synthesized as described previously [17]. On day 13 post surgery, ex vivo autoradiography (AR) was performed on 38 rats 40 min after i.v. injection of approximately 40 MBq ^68^Ga-PSMA or ^18^F-DCFPyL, respectively (Table 1). Five animals for each tracer, and each tumor model were additionally investigated by micro-PET using a small animal Siemens INVEON scanner as described previously [18]. The PET measurement was performed during accumulation phase of the tracers from 20–40 min post injection. For the AR, animals were sacrificed, rat brains removed, and immediately frozen in liquid isopentane (− 50 °C). Every tenth slice of the 20-μm cryosections of the tumor bearing brain and freshly prepared 20 μm ^68^Ga and ^18^F standards, to generate a tracer calibration curve, were exposed to an imaging plate (Fuji Imaging Plate, Raytest) overnight, scanned (Fuji BAS Reader 5000, Raytest), and quantitatively evaluated with a pixel size of 25 μm (AIDA Version 4.50, Raytest). Tracer uptake in the tissue was expressed as standardized uptake value (SUV) by dividing the radioactivity (kBq/ml) in the tissue by the radioactivity injected per gram of body weight.

### Histological staining

Cryosections of the brains were stained with DAPI (4′,6-diamidino-2-phenylindole) and evaluated by fluorescence microscopy. Disturbance of the BBB was visually evaluated using Evans blue dye (EBD). Therefore, 500 μl/kg 2% EBD was injected i.v. 30 min before decapitation of the rat for AR. EBD extravasation in brain slices was examined by fluorescence microscopy (LMD6000, Leica Microsystems CMS GmbH) and processed by AIDA software (AIDA Version 4.50, Raytest).

Blood vessels were stained with anti-rat von Willebrand factor antibody (ab6994, Abcam). To visualize activated microglia, anti-rat CD11b (Integrin alpha M) antibody (ab133357, Abcam) was used. Reactive astrocytes were visualized by staining with anti-rat GFAP (glial fibrillary acidic protein) antibody (ab53554, Abcam). Immunofluorescence PSMA staining was applied to tumor bearing brain slices using three different anti-PSMA antibodies (NBP1-45057 and NBP1-89822, Novus Biologicals; ab58779, Abcam) to display PSMA expression. To verify the functionality and specificity of the used PSMA antibodies, rat prostate and kidney tissue were used as positive controls. Immunofluorescence stainings were performed according to standard histology protocols and as described before [19].

### Pharmacological interventions

Competition tests were performed with four animals to evaluate binding specificity of ^68^Ga-PSMA (*n* = 2) and ^18^F-DCFPyL (*n* = 2). Competitor 30 mg/kg BW 2-(phosphonomethyl)pentane-1,5-dioic acid (PMPA; Tocris) was co-injected i.v. with the tracer as described in the literature [20]. After 40 min of incubation, autoradiography was performed as described above.

In order to evaluate the influence of BBB permeability on tracer uptake, four rats with U87 gliomas received glucocorticoid treatment with dexamethasone (Jenapharm®, Mibe GmbH). The rats received 8 mg/kg intraperitoneally on day 11 post surgery and 4 mg/kg each on days 12 and 13. Autoradiography was performed after injection of ^68^Ga-PSMA as described above.

### Data evaluation

The autoradiograms were co-registered to the DAPI stained sections of adjacent slices to compare tracer binding to morphological data as described previously [19]. In short, a circumference region of interest (ROI) was drawn along the borders of the coronal brain slices in the DAPI stained sections. After adapting the size of the corresponding autoradiogram to that ROI, tracer binding in the tumor region was evaluated by three different ROIs: (1) along the outer tumor margin reflecting the total tumor volume, (2) in the central part of the tumor, and (3) around the peritumoral area. The ROI in the center of the tumor reflects tracer binding in the tumor tissue, excluding the prominent spherical tracer binding in the peritumoral area. The ROI on the peritumoral area contained only the prominent spherical tracer binding, without the binding in the center. Furthermore, a reference ROI was placed in the normal brain tissue, and tumor-to-background ratios (TBR) were calculated by dividing the mean SUV of the different tumor ROIs by the mean SUV of the background ROI. Data evaluation included the tumor volume (mm^3^), SUV in the ROIs, and TBRs.

The evaluation of PET data was performed as described previously [18]. Tumor VOI and contralateral background VOI (110 mm^3^) were placed for each animal in summed PET images from 20 to 40 min p.i. The tumor VOI on PET scans was determined by a 3D autocontouring process using a cutoff for the TBR that yielded a tumor size similar to that calculated in subsequent autoradiography. Afterwards, SUV and TBR were calculated as described above.

### Statistics

Statistical evaluation was performed using Sigma Plot 12.5 (Systat Software GmbH). Two-way repeated measures ANOVAs with Holm-Sidak post-hoc test were performed (1) to compare the volume of total tracer binding in the AR to the histological tumor volume derived from the nuclear staining, (2) to compare tracer uptake in the different tumor models for the AR data, and (3) for the comparison of control animals versus Dex-treated animals. For analysis of astrocytes, comparison of staining intensity between the two treatment groups was performed by *t* test.

## Results

### Autoradiography and micro-PET

**Fig. 1:**
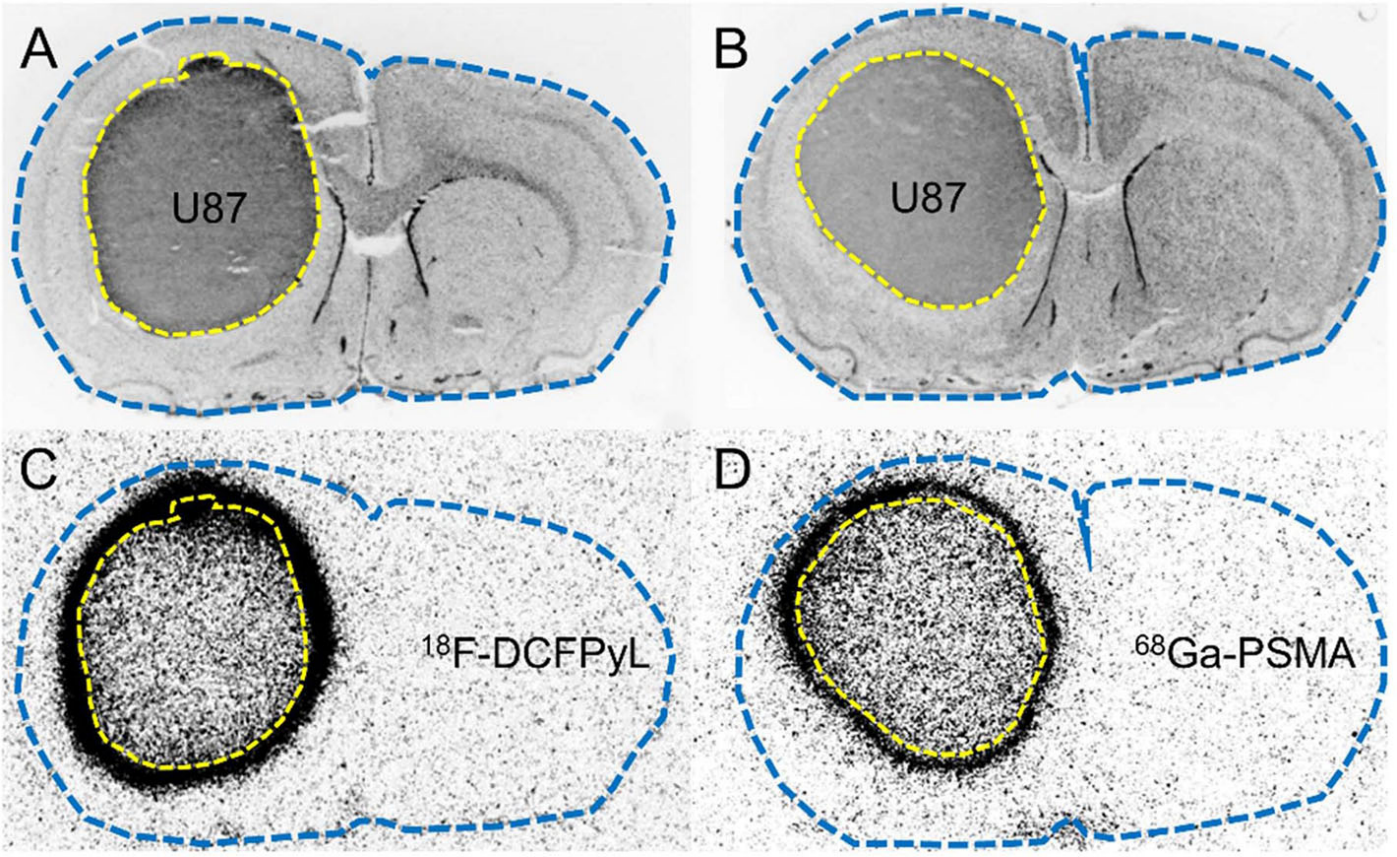
Comparison of histological stainings (nuclear staining with DAPI, **a**, **b**) and autoradiograms (**c**, **d**) of U87 rat gliomas. Tumor tissue is outlined by a dotted yellow line in the histological slices (**a**, **b**) and projected onto the corresponding autoradiograms (**c**, **d**). The outer edge of brain tissue is outlined by a dotted blue line. Both the ^18^F-DCFPyL (**c**) and ^68^Ga-PSMA autoradiograms (**d**) demonstrate prominent uptake in the peritumoral region

Visual evaluation of the autoradiograms of the tumor bearing animals revealed a prominent spherical binding of both ^68^Ga-PSMA and ^18^F-DCFPyL at the rim of the tumors and a lower binding within the center of the tumors in all three tumor models (Fig. 1, suppl. Fig 1). In contrast to the 9 L and U87 tumor models, all F98 tumors showed a central necrosis which showed slightly increased tracer binding. The co-registration of the autoradiographic and histological data demonstrated that the prominent spherical accumulation of the PSMA ligands projected on the peritumoral tissue outside the solid tumor mass. Correspondingly, total tumor volume in autoradiography was significantly larger than histological tumor volume for both tracers in all tumor models (*p* < 0.001) (Table 2).

**Table 2:** Quantification of histological tumor volume (DAPI) and total volume in autoradiography (AR total) for ^18^F-DCFPyL and ^68^Ga-PSMA and all tumor models. Values are given as mean ± SD (*n* = 5 in each group). For both tracers and all tumor models, the autoradiographic volume was significantly larger than the histological tumor volume

	Tracer	Model	DAPI	AR_**total**_	***p*** value
Volume (mm^3^)	^18^F-DCFPyL	U87	53.8 ± 38.3	82.8 ± 52.3	< 0.001
		9 L	105.5 ± 18.7	169.8 ± 27.0	< 0.001
		F98	71.4 ± 13.1	132.9 ± 13.3	< 0.001
	^68^Ga-PSMA	U87	70.1 ± 22.4	95.3 ± 29.4	< 0.001
		9 L	78.6 ± 28.0	107.2 ± 36.0	< 0.001
		F98	58.2 ± 12.0	92.6 ± 8.5	< 0.001

SUV in the center of the tumors was significantly higher for ^68^Ga-PSMA than for ^18^F-DCFPyL in all tumor models. Furthermore, the TBR of tracer uptake at the tumor rim was significantly higher for ^18^F-DCFPyL than for ^68^Ga-PSMA in all tumor models (Table 3). Furthermore, high uptake was noted in the circumventricular organs, namely, the organum vasculosum of the lamina terminalis (suppl. Fig 1), the median eminence, and the subfornical organ (data not shown).

**Table 3:** Tracer uptake in autoradiography for all glioma models quantified by SUV (mean ± SD) and tumor-to-brain ratios (TBR, mean ± SD). SUV in the center of the tumors was significantly higher for ^68^Ga-PSMA than for ^18^F-DCFPyL in all tumor models. Furthermore, the TBR of tracer uptake at the tumor rim was significantly higher for ^18^F-DCFPyL than for ^68^Ga-PSMA in all tumor models. The differences have to be interpreted carefully, since the data may be influenced by the different spatial resolution when using ^68^Ga or ^18^F

Model	Parameter	Region	^**68**^Ga-PSMA	^**18**^F-DCFPyL	***p*** value
U87	SUV	Tumor center	0.39 ± 0.09	0.22 ± 0.08	0.005
		Tumor rim	0.77 ± 0.13	0.87 ± 0.13	n.s.
		Brain	0.016 ± 0.004	0.004 ± 0.002	n.s.
	TBR	Tumor center	25.1 ± 2.1	58.4 ± 13.1	n.s.
		Tumor rim	50.0 ± 13.1	256.2 ± 27.9	< 0.001
9 L	SUV	Tumor center	0.85 ± 0.08	0.37 ± 0.20	< 0.001
		Tumor rim	1.31 ± 0.28	0.85 ± 0.23	< 0.001
		Brain	0.013 ± 0.001	0.006 ± 0.004	n.s.
	TBR	Tumor center	64.43 ± 6.65	69.86 ± 19.86	n.s.
		Tumor rim	99.91 ± 23.17	181.17 ± 81.14	0.011
F98	SUV	Tumor center	0.43 ± 0.07	0.28 ± 0.02	0.006
		Tumor rim	0.78 ± 0.16	0.78 ± 0.09	n.s.
		Brain	0.013 ± 0.001	0.006 ± 0.002	n.s.
	TBR	Tumor center	34.10 ± 7.04	50.48 ± 20.79	n.s.
		Tumor rim	62.39 ± 16.57	139.84 ± 48.27	0.001

**Fig. 2:**
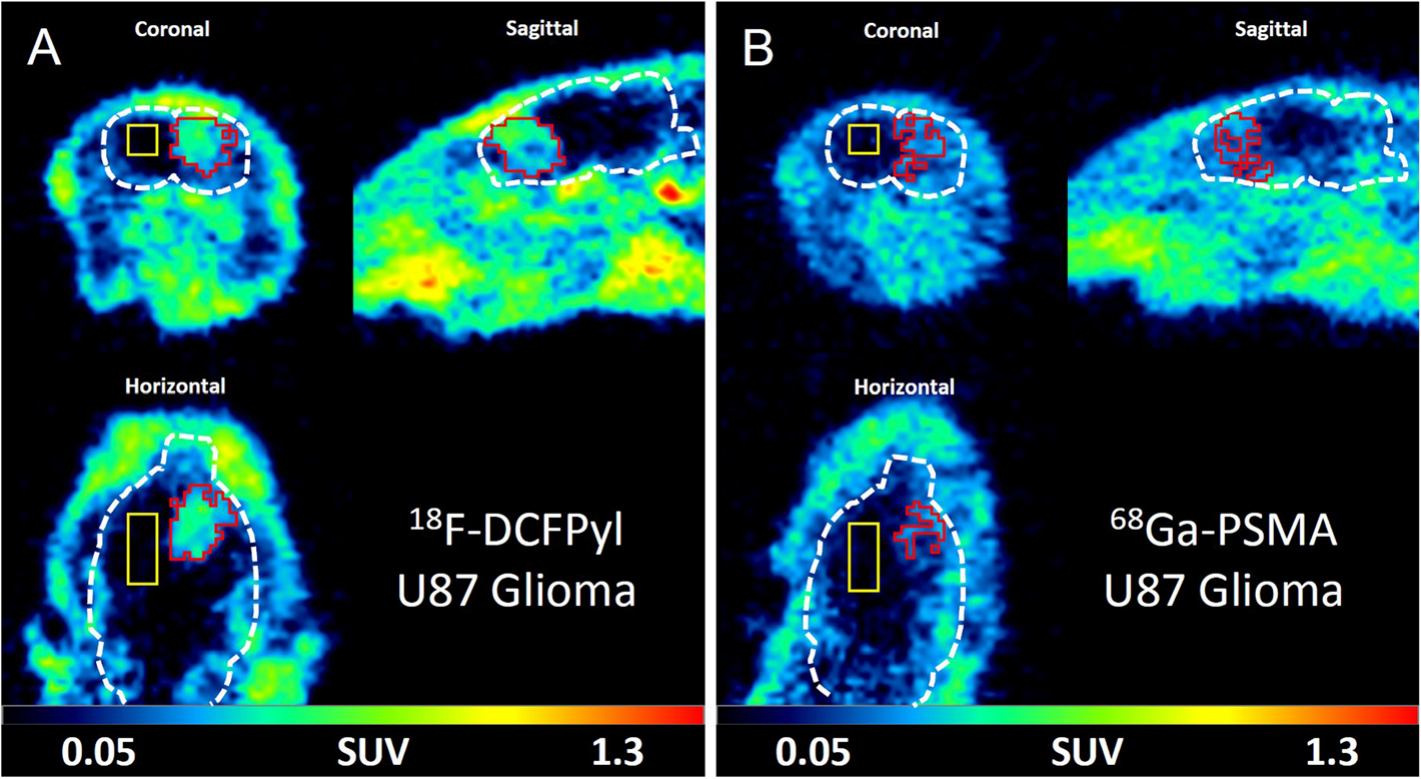
Coronal, sagittal, and horizontal micro-PET images of the rat brain with intracerebral U87 glioma for ^18^F-DCFPyL (**a**) and ^68^Ga-PSMA (**b**) (summed PET images from 20 to 40 min postinjection). Brain is outlined by a white dotted line. Tumor VOI is indicated by a red line and brain VOI (110 mm^3^) in the contralateral hemisphere by a yellow line. Tumor visualization is considerably better with ^18^F-DCFPyL than with ^68^Ga-PSMA

The PET images showed a higher tumor to brain contrast for ^18^F-DCFPyL than for ^68^Ga-PSMA allowing a more precise estimation of tumor location for all tumor models (Fig. 2, suppl. Fig. 2 and 3). The lower spatial resolution of the PET scans did not allow the identification of the high, spherical accumulation of ligands in the periphery of the tumors. The quantitative analysis of tracer uptake within tumor VOIs showed no significant differences for tumor SUVs between both tracers for all three tumor models while SUV in the brain tissue was significantly lower for ^18^F-DCFPyL than for ^68^Ga-PSMA. This led to significantly higher TBRs for ^18^F-DCFPyL compared with ^68^Ga-PSMA (Table 4).

**Table 4:** ^68^Ga-PSMA and ^18^F-DCFPyL uptake in tumor and contralateral brain in micro-PET for U87, 9 L, and F98 models (each group *n* = 5) quantified by SUV and TBR (mean ± SD). SUV in the brain was significantly higher for ^68^Ga-PSMA than for ^18^F-DCFPyL in all tumor models leading to higher TBR for ^18^F-DCFPyL in all tumor models. The differences have to be interpreted carefully, since the data are influenced by the different spatial resolution when using ^68^Ga or ^18^F

Model	Parameter	Region	[^**68**^Ga]PSMA	[^**18**^F] DCFPyL	***p*** value
**U87**	**SUV**	Tumor	0.31 ± 0.05	0.32 ± 0.07	n.s.
		Brain	0.10 ± 0.02	0.05 ± 0.01	< 0.001
	**TBR**		3.26 ± 0.55	6.28 ± 0.68	< 0.001
**9 L**	**SUV**	Tumor	0.46 ± 0.09	0.56 ± 0.11	n.s.
		Brain	0.12 ± 0.01	0.07 ± 0.02	< 0.001
	**TBR**		3.92 ± 0.57	7.92 ± 1.99	< 0.001
**F98**	**SUV**	Tumor	0.39 ± 0.05	0.44 ± 0.04	n.s.
		Brain	0.12 ± 0.02	0.07 ± 0.01	< 0.001
	**TBR**		3.22 ± 0.50	6.85 ± 1.33	< 0.001

### Pharmacological interventions

**Fig. 3:**
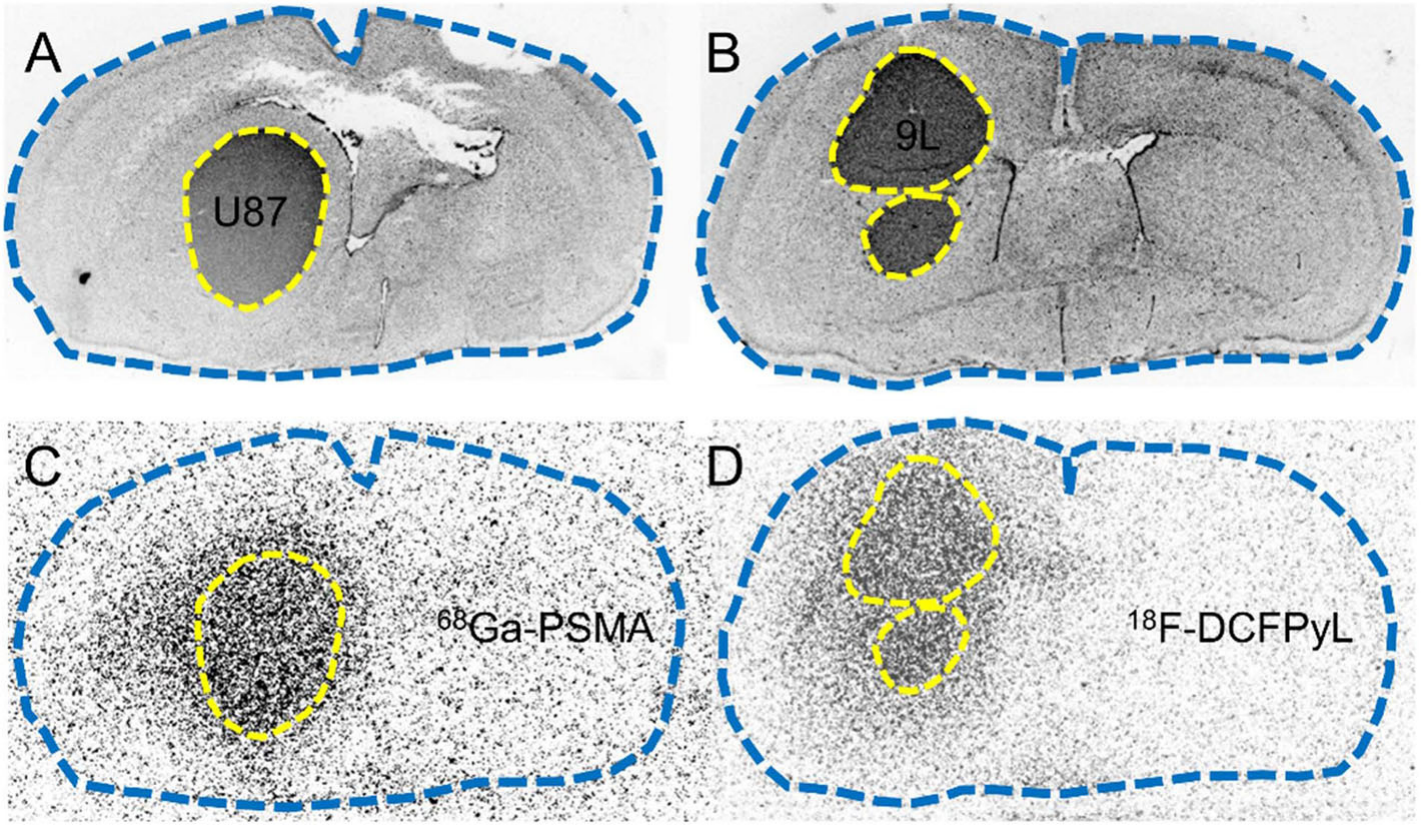
Influence of PMPA competition on ^68^Ga-PSMA and ^18^F-DCFPyL uptake in U87 (left) and 9 L glioma (right), respectively. Coronal brain slices of histological staining (**a**, **b**) and autoradiography (**c**, **d**). Tumor tissue is outlined as a dotted yellow line in the nuclear staining and projected onto the autoradiograms, and the outer edge of brain tissue is outlined by a blue dotted line. Co-injection of PMPA blocks ^68^Ga-PSMA uptake in the periphery of the 9 L glioma (**c**) and ^18^F-DCFPyL uptake in U87 glioma (**d**)

Competition experiments using the PSMA specific ligand PMPA led to a disappearance of the increased spherical tracer binding in the peritumoral area (Fig. 3), and the TBR of ^68^Ga-PSMA and ^18^F-DCFPyL uptake decreased considerably with PMPA (suppl. Tab. 1). Furthermore, tracer uptake in the circumventricular organs was completely supressed by PMPA treatment (data not shown).

**Fig. 4:**
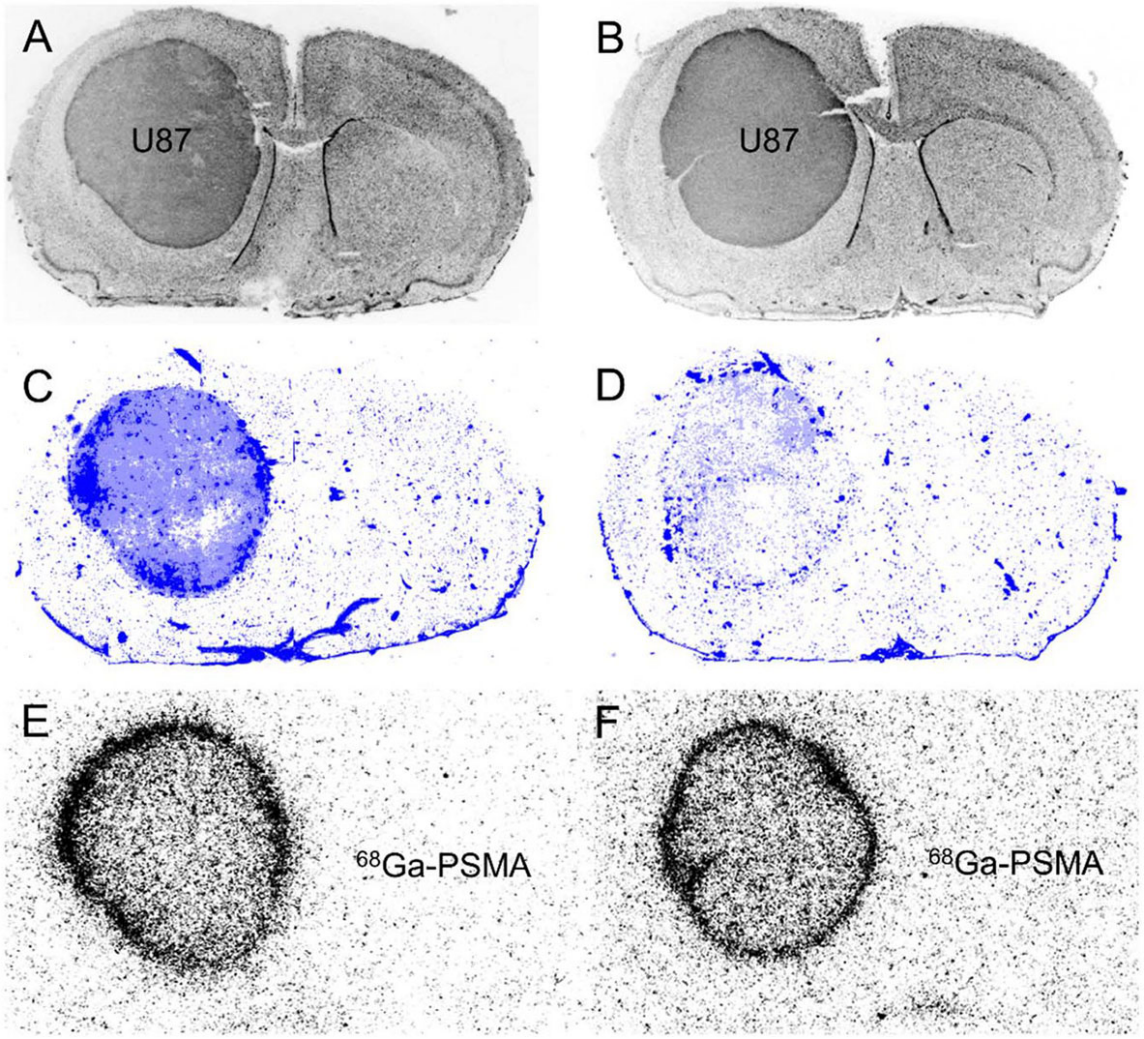
Coronal brain slices of an untreated (left column) and dexamethasone (Dex)-treated U87 bearing rat (right column). Histological staining (DAPI) (**a**, **b**), Evans blue staining (**c**, **d**) and ^68^Ga-PSMA autoradiography (**e**, **f**). The untreated animal shows a strong extravasation of Evans blue into the tumor tissue indicating BBB disruption (**c**). In contrast, the Dex-treated animal shows little extravasation of Evans blue into tumor tissue indicating a restoration of the BBB after Dex treatment (**d**). ^68^Ga-PSMA autoradiography shows an unchanged prominent spherical tracer binding at the tumor rim both in the untreated animal (**e**) and after Dex treatment (**f**)

Dex treatment of U87 glioma bearing animals led to an almost complete reduction of EBD extravasation, indicating a restoration of the BBB or at least drastic reduction of BBB permeability (Fig. 4). In contrast, ^68^Ga-PSMA uptake showed no significant difference in the untreated and Dex-treated U87 gliomas indicating that the change of BBB permeability had only a minor influence on tracer uptake in the tumors (Table 5).

**Table 5:** Tracer uptake for ^68^Ga-PSMA in different regions of dexamethasone-treated (*n* = 5) and untreated (*n* = 5) U87 tumors quantified by SUV (mean ± SD) and tumor-to-brain ratios (TBR, mean ± SD). There was no significant difference in ^68^Ga-PSMA uptake in the tumor after dexamethasone treatment

Parameter	Region	Treated	Untreated	***p*** value
SUV	Tumor center	0.35 ± 0.04	0.39 ± 0.09	0.339
	Tumor rim	0.74 ± 0.05	0.77 ± 0.13	0.598
	Brain	0.017 ± 0.004	0.015 ± 0.004	0.976
TBR	Tumor center	20.69 ± 5.14	25.09 ± 2.11	0.438
	Tumor rim	43.44 ± 7.27	50.02 ± 13.11	0.253

### PSMA staining

**Fig. 5:**
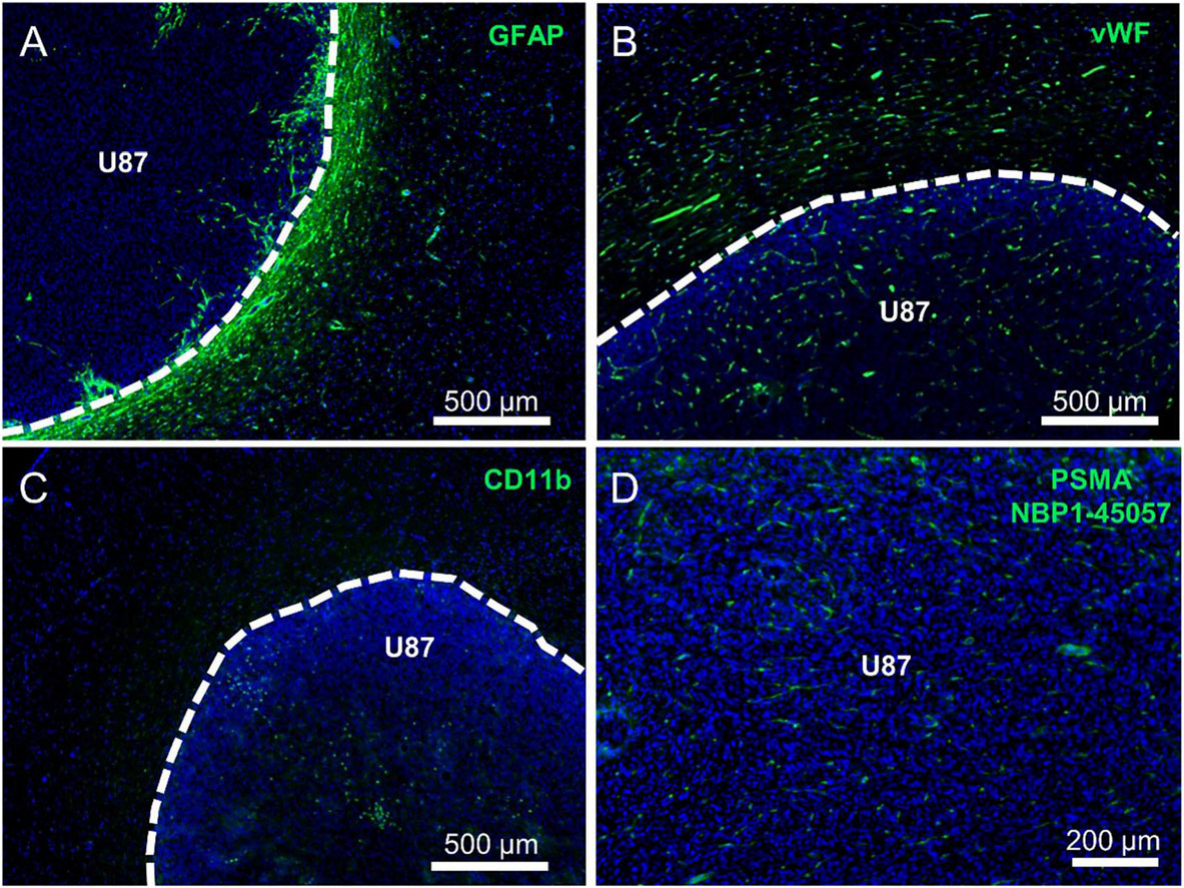
Immunofluorescence staining of U87 tumors. Nuclear staining (DAPI) is shown in blue, antibody staining in green, and tumor borders are outlined in white. Strong reactive astrocytosis (GFAP; **a**) is visible in the peritumoral region with few astrocytes at the tumor border. Specific staining of blood vessels (vWF; **b**) is detectable, which is similar within tumor tissue and in the peritumoral region. Few activated microglia (CD11b; **c**) can be observed in the peritumoral region and inside the tumor. PSMA staining using the antibody NBP1-45057 is positive in vessel-like structures in the tumor center (**d**) but not outside the tumor

PSMA expression in the tumor and the peritumoral area was tested by three different PSMA antibodies. The antibody ab58779 demonstrated no staining in the peritumoral or the tumor center area while rat prostate and kidney tissue showed a positive staining (data not shown). The antibody NBP1-89822 showed positive staining of 9 L and F98 tumor cells (suppl. Fig. 4), but no staining in the peritumoral region. U87 tumors were negative (data not shown). The antibody NBP1-45057 showed a positive staining on vessel-like structures in all glioma models (Fig. 5; suppl. Fig. 4) but no staining in the peritumoral area.

### Staining with cellular markers

The vasculature of the tumors was stained with the anti-von Willebrand factor antibody, which visualized dense and enlarged vessels inside the tumor and in the peritumoral area of all glioma models (Fig. 5; suppl. Fig. 4). There was no evidence of a higher density of blood vessels in the peritumoral area or at the tumor border in comparison to the main tumor mass.

CD11b staining demonstrated low presence of activated microglia in the tumor and/or the peritumoral area (Fig. 5; suppl. Fig. 4).

In contrast, GFAP staining of reactive astrocytes showed a strong signal in the peritumoral area in all tumor models in congruence with the prominent spherical binding of ^68^Ga-PSMA and ^18^F-DCFPyL. Very few astrocytes were noted in the center of the tumors. An example of GFAP staining of U87 glioma is shown in Fig. 5, for 9 L and F98 glioma in suppl. Fig. 4.

## Discussion

In recent years, PET imaging using PSMA-selective tracers like ^68^Ga-PSMA has been shown to be a very sensitive and specific method for the diagnosis of prostate cancer, as PSMA is highly expressed in prostate carcinoma cells [21]. High PSMA expression, however, has also been shown in the neovasculature of solid tumors, including gliomas, making this a promising approach for brain tumor targeting [10, 11]. Recent studies reported on high binding of ^68^Ga-PSMA and ^18^F-DCFPyL in human brain tumors, and presence of PSMA was immunohistochemically identified either in tumor cells or in neovasculature of these tumors [13–15]. However, direct binding of PSMA ligands to a specific cell population within the tumor tissue has not been proven so far. In addition, it remains unclear to what extent BBB permeability influences the uptake of PSMA ligands in gliomas. Therefore, we analyzed the ^68^Ga-PSMA and ^18^F-DCFPyL distribution in different rat gliomas using autoradiography in comparison to various histological stainings. Furthermore, the specificity of tracer binding and the role of BBB permeability were addressed.

Surprisingly, autoradiography revealed a prominent spherical uptake of ^68^Ga-PSMA and ^18^F-DCFPyL around the U87, 9 L, and F98 gliomas, which was similar for both tracers (Fig. 1, suppl. Fig 1). Comparison with histological staining revealed that the tracer binding clearly projected outside the solid tumor mass in the peritumoral region. Previous PET studies in glioma patients did not report such findings, which may be explained by the considerably lower spatial resolution of PET compared with the ex vivo autoradiography used here. Accordingly, the micro-PET examinations in our study were not able to detect the spherical accumulation of the PSMA ligands in the periphery of the tumors.

Tracer binding in the tumors was further analyzed by competition experiments using the PSMA inhibitor PMPA [22]. PMPA is highly specific to PSMA, which has been demonstrated by lack of binding to more than 100 different receptors, ion channels, transporters, and enzymes [23]. PMPA as well as the PSMA-selective tracers bind to the active site on the extracellular part of the transmembrane protein PSMA. The co-injection of PMPA led to a complete suppression of ^68^Ga-PSMA and ^18^F-DCFPyL binding in the peritumoral areas of U87 and 9 L gliomas, respectively (Fig. 3), and to a reduction of tracer uptake in the center of the tumors. These findings strongly suggest that the observed phenomenon of tracer uptake in the periphery of the tumors reflects specific binding to PSMA receptors.

The influence of BBB permeability on tracer uptake was investigated by treatment with the glucocorticoid Dex as described previously [24]. A partial restoration of BBB of the tumors by Dex treatment led to a considerable decrease of Evans blue extravasation into the tumor tissue of Dex-treated rats. In contrast, no significant differences of ^68^Ga-PSMA uptake between untreated and Dex-treated U87 gliomas were observed, in particular at the rim of the tumors, suggesting that the influence of BBB permeability on tracer binding, especially in the periphery of the tumors, is small. This finding is surprising and in contrast to the observation that accumulation of PSMA ligands is usually observed only in brain areas with BBB disruption. Therefore, this finding needs further investigation and confirmation.

Immunofluorescence staining using three different anti-PSMA antibodies showed signals on tumor cells in 9 L and F98 gliomas and on vessel-like structures in U87, 9 L, and F98 gliomas (Fig. 5, suppl. Fig. 4) similar to observations in human gliomas [10, 11]. However, no PSMA staining was observed in the peritumoral region. One possible explanation for this finding is the existence of different PSMA isoforms which are present in the brains of rats due to different splice variants of the FOLH1 gene [11, 25, 26] that are not recognized by the antibodies used. Little is known about PSMA isoforms in rats, and information about specificity of the used antibodies for different rat PSMA isoforms is not available leaving this question open.

Vascular staining using an antibody against von Willebrand factor showed similar immunofluorescence intensity in the tumor and in the peritumoral area (Fig. 5, suppl. Fig 4), which virtually excludes that differences in neovascularization can explain the prominent tracer binding in the periphery of the tumors. Furthermore, evaluation of microglial activation by CD11b staining demonstrated only a few microglia cells in the peritumoral area. Therefore, it can be assumed that the tracer binding in this area is not caused by microglial activation around the tumor (Fig. 5, suppl. Fig. 4).

The examination of the tumor bearing brain slices for activated astrocytes using anti-GFAP staining, however, revealed a strong staining in the peritumoral area of all tumor models (Fig. 5, suppl. Fig 4) which was similar to the pattern of tracer binding at the circumference of the tumors. This observation suggests that the pronounced accumulation of PSMA ligands in the periphery of tumors is associated with reactive astrocytosis. Astrocytes are the most abundant glial cell type in the brain and are involved in many processes such as regulation of brain homeostasis, maintenance, and repair of the BBB and neurogenesis [27–29]. Astrocytes are capable of actively reacting to different kinds of neurological disorders by hypertrophy and change of morphology and functions [30]. This process is referred to as reactive astrocytosis and includes the upregulation of GFAP, the main intermediate filament of the astrocytes. In the presence of tumor cells, astrocytes are activated and tend to surround gliomas, exerting both anti-tumoral and pro-tumoral functions [31]. A high expression of PSMA, also known as glutamate carboxypeptidase II (GCP II), has been reported in astrocytes of the rat brain [32]. Furthermore, investigations of human brain tissue revealed that astrocytes specifically express GCP II in all parts of the human brain [33]. Thus, it seems very likely that the here observed intense accumulation of PSMA ligands in the periphery of all tumor models is caused by binding to reactive astrocytes. The hypothesis is further supported by a recent case report, which observed an accumulation of ^18^F-DCFPyL in a radionecrosis after treatment of a brain metastasis [34]. Since reactive astrocytosis typically occurs in radionecrosis [35], binding of the PSMA ligand to reactive astrocytes would well explain the supposed false positive finding.

The comparison of ^68^Ga-PSMA and ^18^F-DCFPyL showed differences in uptake at the tumor center and TBR at the tumor margin in all tumor models (Table 3), and also different TBRs in micro-PET (Table 4). These differences must be interpreted carefully, since positron range effects of the two different isotopes can influence the data. The imaging characteristics of positron emitters with different β + energies on autoradiography using phosphor imaging plates have been analyzed in a previous study [36]. In that study using imaging plates similar to that in our study (BAS-SR 2025), the spatial resolution of ^18^F (E_max_ 0.63 MeV) was 339 ± 24 μm (FWHM) and that of ^15^O (E_max_ 1.73 MeV), which is close to that of ^68^Ga (E_max_ 1.899 MeV), was 420 ± 72 μm (FWHM). Thus, the differences may partly be explained by the lower spatial resolution when using ^68^Ga. For micro-PET, the spatial resolution for ^18^F in water is 2.0 mm (FWHM) versus 2.8 mm for ^68^Ga leading to similar effects [37].

The study is limited by the fact that the specific binding of PSMA ligands to reactive astrocytes could not be documented by corresponding immunostaining using PSMA antibodies. This requires further experiments with different antibodies recognizing specific PSMA isoforms. Furthermore, it is questionable whether the results of the animal experiments are transferable to humans. This is at least supported by the detection of PSMA on human astrocytes as well as the observation of unspecified accumulation of PSMA ligands in a case of radionecrosis as described above.

## Conclusions

The results of this study strongly suggest that reactive astrogliosis can lead to an accumulation of PSMA ligands that significantly exceeds the binding to the actual tumor tissue. This observation could severely limit the validity of the method for assessing brain tumor tissue in patients. In particular, this result raises doubts as to whether the method is suitable for theranostics.

## Supplementary information

**Additional file 1 Figure 1:** Comparison of histological stainings (DAPI, A, C, E, G) and autoradiograms (B, D, F, H) of 9L (A – D) and F98 (E – H) rat gliomas with corresponding ^18^F-DCFPyL and ^68^Ga-PSMA autoradiograms. Tumor tissue is outlined by a dotted yellow line in the histological slices and projected onto the autoradiogram. The outer edge of brain tissue is outlined by a dotted blue line. Tracer binding is similar for both tracers with prominent uptake in the peritumoral region. The F98 tumors exhibit a central necrosis with increased uptake (E-H). The organum vasculosum of the lamina terminalis (arrow in B), one of the circumventricular organs lacking a blood-brain barrier, exhibits also high uptake. **Figure 2:** Coronal, sagittal and horizontal micro-PET images of the rat brain with intracerebral 9L glioma for ^18^F-DCFPyL (A) and ^68^Ga-PSMA (B) (summed PET images from 20 to 40 min postinjection). Brain is outlined by a white dotted line. Tumor VOI is indicated by a red line and brain VOI (110 mm^3^) in the contralateral hemisphere by a yellow line. Tumor visualization is considerably better with ^18^F-DCFPyL than with ^68^Ga-PSMA. **Figure 3:** Coronal, sagittal and horizontal micro-PET images of the rat brain with intracerebral F98 glioma for ^18^F-DCFPyL (A) and ^68^Ga-PSMA (B) (summed PET images from 20 to 40 min postinjection). Brain is outlined by a white dotted line. Tumor VOI is indicated by a red line and brain VOI (110 mm^3^) in the contralateral hemisphere by a yellow line. Again, tumor visualization is considerably better with ^18^F-DCFPyL than with ^68^Ga-PSMA. **Table 1:** Competition of tracer binding with PMPA. Comparison between tracer binding in rats injected with PSMA tracer in the presence (w/ PMPA) or absence of PMPA (w/o PMPA) for the pattern of the tumor area and the contralateral brain region (mean values +/- SD). **Figure 4:** Immunofluorescence staining of 9L tumors (left column) and F98 tumors (right column). Nuclear staining (DAPI) is shown in blue, antibody staining in green, and tumor borders are outlined in white. Strong reactive astrocytosis (GFAP; A, F) is visible in the peritumoral region with few astrocytes at the inner tumor border. A few activated microglia (CD11b; B, G) can be observed in the peritumoral region of the 9L and F98 tumor as well as within the tumor tissue of F98. Specific staining of blood vessels (vWF; C, H) is visible within tumor tissue and in the peritumoral region of 9L and F98 tumors. No higher vascularization in the peritumoral region in relation to the tumor tissue was found. Specific staining of PSMA using the antibody NBP1-45057 is visible within the tumor center of 9L and F98 (D, I) but not outside the tumor, revealing vessel-like structures. Specific staining of PSMA using the antibody NBP1-89822 (E, J) is visible within 9L tissue and seems to be located around the tumor cell nuclei. F98 tissue shows fewer positive signals in comparison with 9L. No staining was observed in the peritumoral region, independent of the tumor model.

## Data Availability

The datasets used and/or analyzed during the current study are available from the corresponding author on reasonable request**.**

## Abbreviations

ANOVA: Analysis of variance
AR: Autoradiography
BBB: Blood-brain barrier
CD11b: Antibody against activated microglia
DAPI: 4′,6-Diamidino-2-phenylindole
Dex: Dexamethasone
DMEM: Dulbecco’s Modified Eagle’s Medium
EBD: Evans blue dye
^18^F-DCFPyL: 2-(3-(1-carboxy-5-[(6-[^18^F]fluoro-pyridine-3-carbonyl)-amino]-pentyl)-ureido)-pentanedioic acid
GFAP: Glial fibrillary acidic protein
MEM: Minimum Essential Medium Eagle
PMPA: 2-(Phosphonomethyl)pentane-1,5-dioic acid; ^68^Ga-PSMA: ^68^Ga-Glu-NH-CO-NH-Lys(Ahx)-HBED-CC
PSMA: Prostate specific membrane antigen
ROI: Regions of interest
SUV: Standardized uptake value
TBR: Tumor-to-background ratio
