# Supplementary material for: High uptake of 68Ga-PSMA and 18F-DCFPyL in the peritumoral area of rat gliomas due to activated astrocytes
Source: EJNMMI Res. 2020 May 25;10:55. doi: 10.1186/s13550-020-00642-0 (PMC7378136; doi:10.1186/s13550-020-00642-0)
Supplement: Supplementary file 1 — Additional file 1 Figure 1: Comparison of histological stainings (DAPI, A, C, E, G) and autoradiograms (B, D, F, H) of 9L (A – D) and F98 (E – H) rat gliomas with corresponding 18F-DCFPyL and 68Ga-PSMA autoradiograms. Tumor tissue is outlined by a dotted yellow line in the histological slices and projected onto the autoradiogram. The outer edge of brain tissue is outlined by a dotted blue line. Tracer binding is similar for both tracers with prominent uptake in the peritumoral region. The F98 tumors exhibit a central necrosis with increased uptake (E-H). The organum vasculosum of the lamina terminalis (arrow in B), one of the circumventricular organs lacking a blood-brain barrier, exhibits also high uptake. Figure 2: Coronal, sagittal and horizontal micro-PET images of the rat brain with intracerebral 9L glioma for 18F-DCFPyL (A) and 68Ga-PSMA (B) (summed PET images from 20 to 40 min postinjection). Brain is outlined by a white dotted line. Tumor VOI is indicated by a red line and brain VOI (110 mm3) in the contralateral hemisphere by a yellow line. Tumor visualization is considerably better with 18F-DCFPyL than with 68Ga-PSMA. Figure 3: Coronal, sagittal and horizontal micro-PET images of the rat brain with intracerebral F98 glioma for 18F-DCFPyL (A) and 68Ga-PSMA (B) (summed PET images from 20 to 40 min postinjection). Brain is outlined by a white dotted line. Tumor VOI is indicated by a red line and brain VOI (110 mm3) in the contralateral hemisphere by a yellow line. Again, tumor visualization is considerably better with 18F-DCFPyL than with 68Ga-PSMA. Table 1: Competition of tracer binding with PMPA. Comparison between tracer binding in rats injected with PSMA tracer in the presence (w/ PMPA) or absence of PMPA (w/o PMPA) for the pattern of the tumor area and the contralateral brain region (mean values +/- SD). Figure 4: Immunofluorescence staining of 9L tumors (left column) and F98 tumors (right column). Nuclear staining (DAPI) is shown in blue, antibody stain [file 13550_2020_642_MOESM1_ESM.docx]

**Supplemental Material**

**
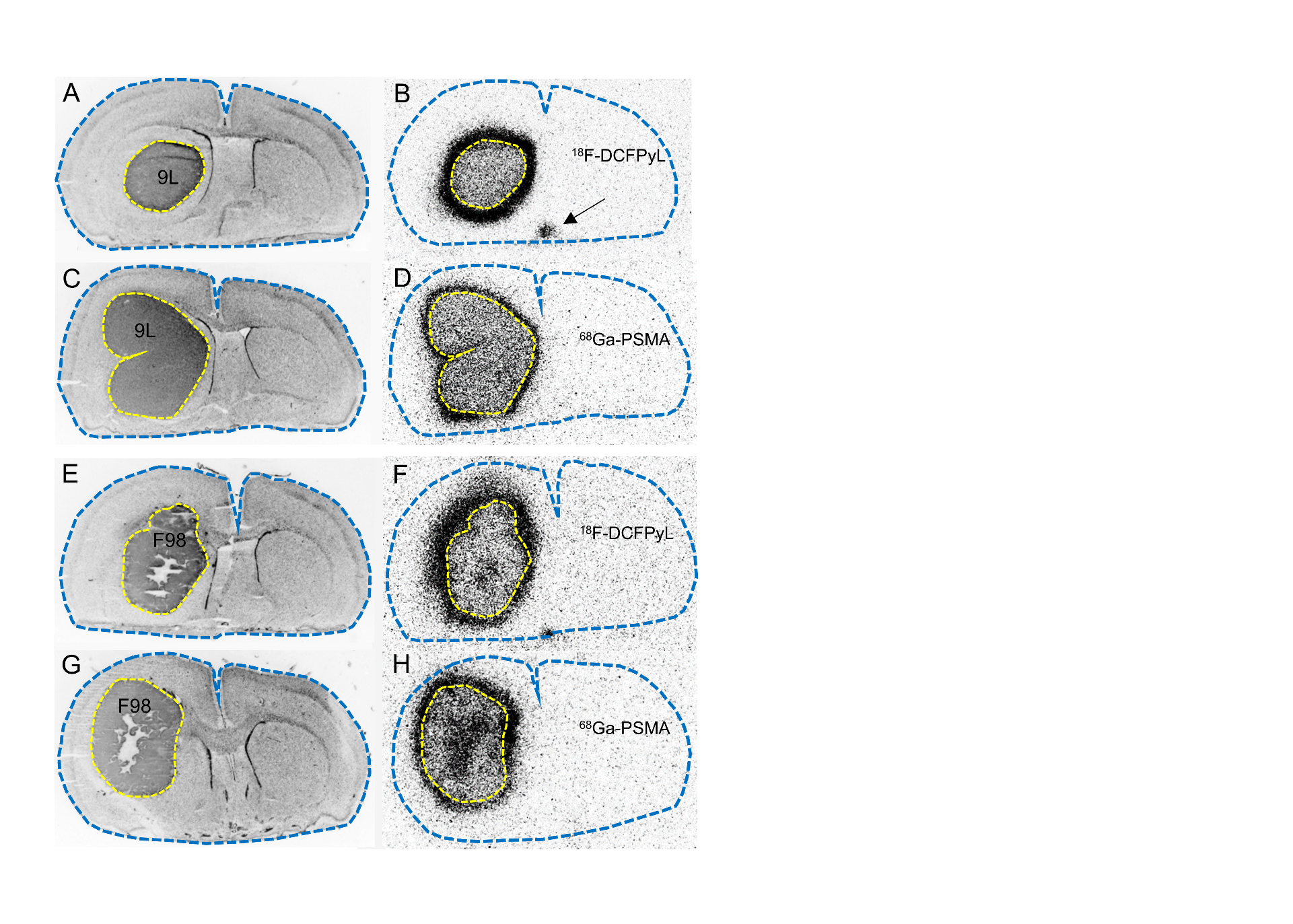
**

**Supplemental Figure 1:** Comparison of histological stainings (DAPI, A, C, E, G) and autoradiograms (B, D, F, H) of 9L (A – D) and F98 (E – H) rat gliomas with corresponding ^18^F-DCFPyL and ^68^Ga-PSMA autoradiograms. Tumor tissue is outlined by a dotted yellow line in the histological slices and projected onto the autoradiogram. The outer edge of brain tissue is outlined by a dotted blue line. Tracer binding is similar for both tracers with prominent uptake in the peritumoral region. The F98 tumors exhibit a central necrosis with increased uptake (E-H). The organum vasculosum of the lamina terminalis (arrow in B), one of the circumventricular organs lacking a blood-brain barrier, exhibits also high uptake.


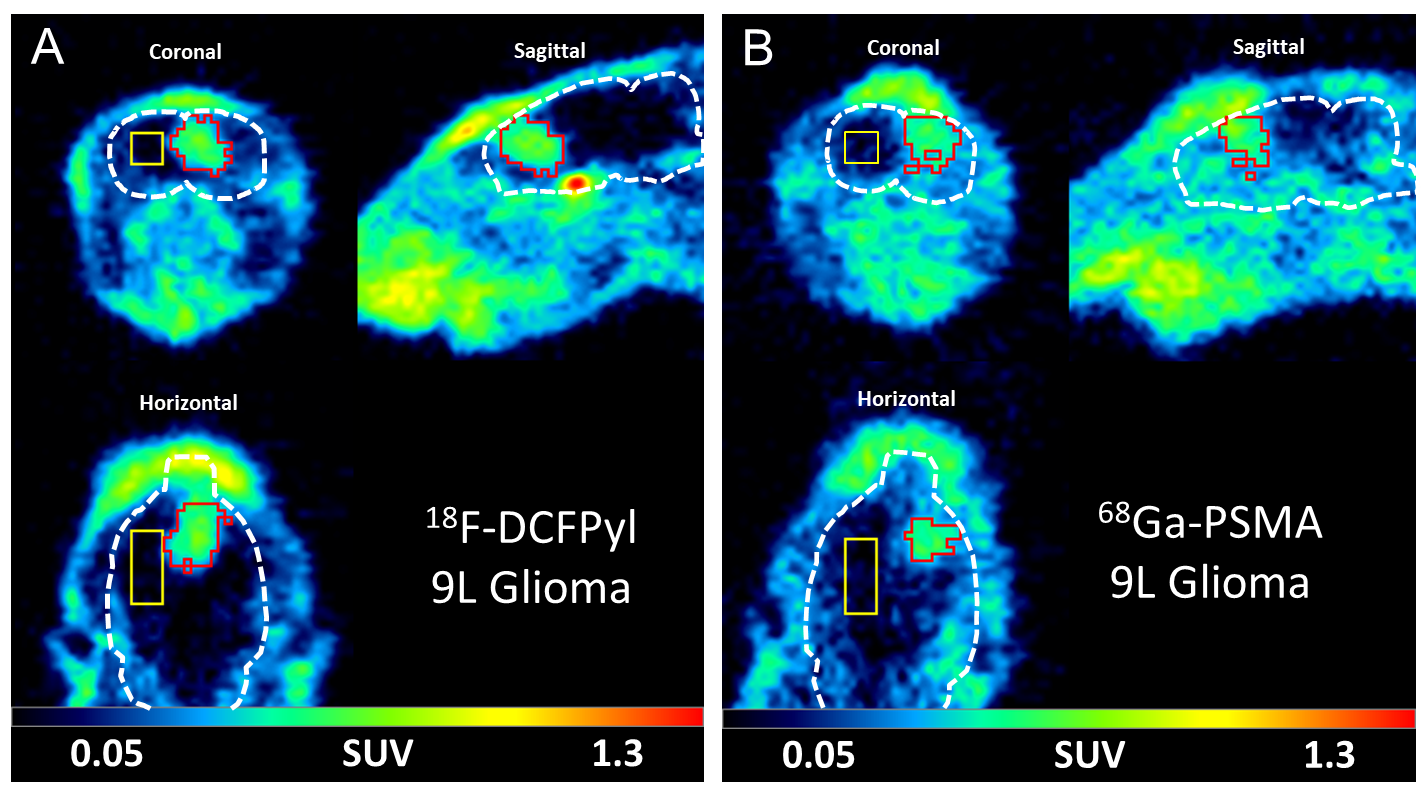


**Supplemental Figure 2:** Coronal, sagittal and horizontal micro-PET images of the rat brain with intracerebral 9L glioma for ^18^F-DCFPyL (A) and ^68^Ga-PSMA (B) (summed PET images from 20 to 40 min postinjection). Brain is outlined by a white dotted line. Tumor VOI is indicated by a red line and brain VOI (110 mm³) in the contralateral hemisphere by a yellow line. Tumor visualization is considerably better with ^18^F-DCFPyL than with ^68^Ga-PSMA.

**Supplemental Figure 3:** Coronal, sagittal and horizontal micro-PET images of the rat brain with intracerebral F98 glioma for ^18^F-DCFPyL (A) and ^68^Ga-PSMA (B) (summed PET images from 20 to 40 min postinjection). Brain is outlined by a white dotted line. Tumor VOI is indicated by a red line and brain VOI (110 mm³) in the contralateral hemisphere by a yellow line. Again, tumor visualization is considerably better with ^18^F-DCFPyL than with ^68^Ga-PSMA.

| **Model/tracer** | **Parameter** | **Region** | **w/ PMPA** | **w/o PMPA** |
| --- | --- | --- | --- | --- |
| U87  ^68^Ga-PSMA | SUV | Tumor | 0.37 ± 0.01 | 0.54 ± 0.09 |
|  |  | Contralateral | 0.016 ± 0.0001 | 0.016 ± 0.004 |
|  | TBR | Tumor | 23.15 ± 0.25 | 34.59 ± 3.85 |
| 9L  ^18^F-DCFPyL | SUV | Tumor | 0.37 ± 0.01 | 0.54 ± 0.19 |
|  |  | Contralateral | 0.01 ± 0.002 | 0.006 ± 0.004 |
|  | TBR | Tumor | 38.81 ± 1.13 | 110.92 ± 43.57 |

**Supplemental Table 1**: Competition of tracer binding with PMPA. Comparison between tracer binding in rats injected with PSMA tracer in the presence (w/ PMPA) or absence of PMPA (w/o PMPA) for the pattern of the tumor area and the contralateral brain region (mean values +/- SD).


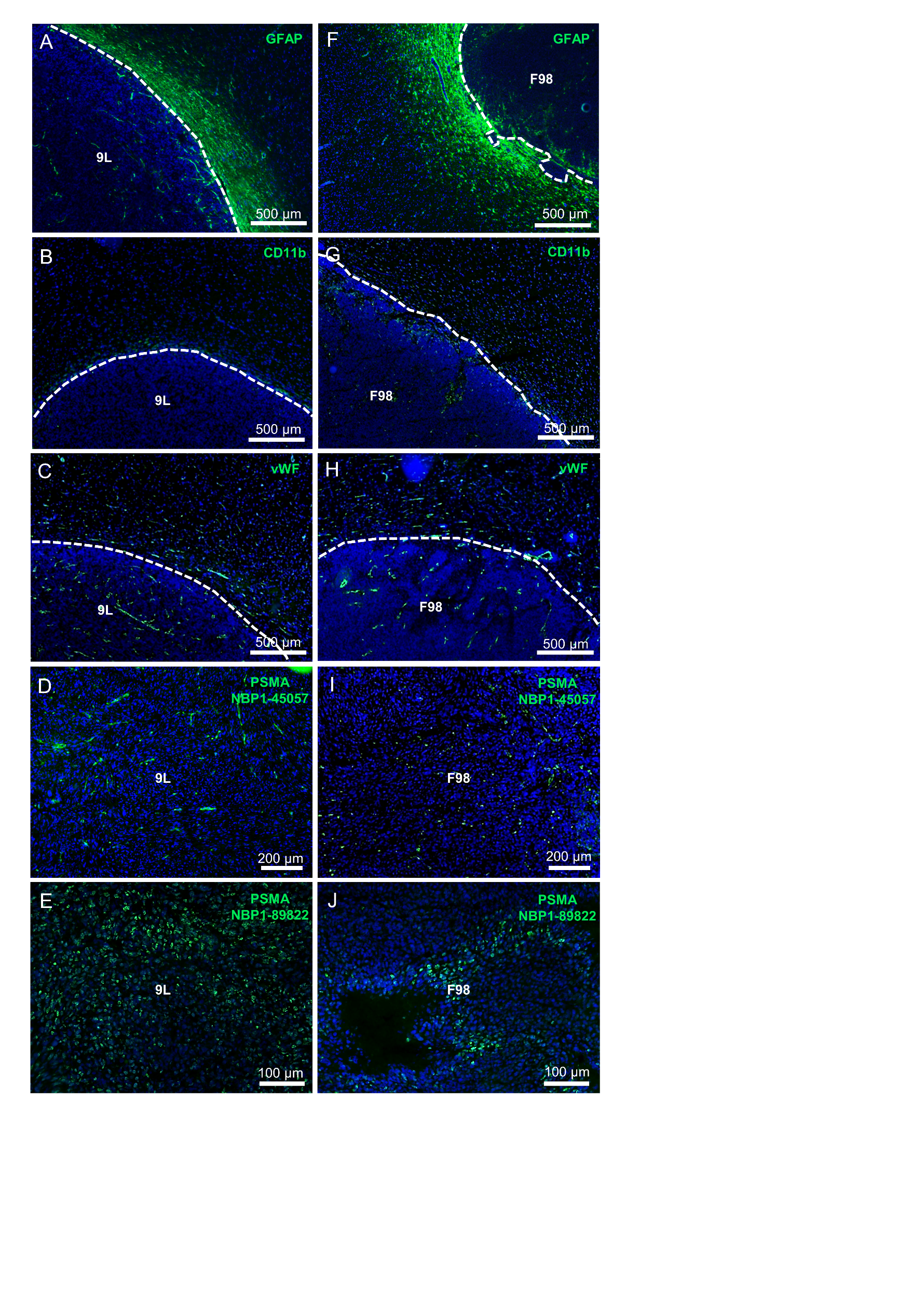


**Supplemental Figure 4:** Immunofluorescence staining of 9L tumors (left column) and F98 tumors (right column). Nuclear staining (DAPI) is shown in blue, antibody staining in green, and tumor borders are outlined in white. Strong reactive astrocytosis (GFAP; A, F) is visible in the peritumoral region with few astrocytes at the inner tumor border. A few activated microglia (CD11b; B, G) can be observed in the peritumoral region of the 9L and F98 tumor as well as within the tumor tissue of F98. Specific staining of blood vessels (vWF; C, H) is visible within tumor tissue and in the peritumoral region of 9L and F98 tumors. No higher vascularization in the peritumoral region in relation to the tumor tissue was found. Specific staining of PSMA using the antibody NBP1-45057 is visible within the tumor center of 9L and F98 (D, I) but not outside the tumor, revealing vessel-like structures. Specific staining of PSMA using the antibody NBP1-89822 (E, J) is visible within 9L tissue and seems to be located around the tumor cell nuclei. F98 tissue shows fewer positive signals in comparison with 9L. No staining was observed in the peritumoral region, independent of the tumor model.
